# Immortal Time Bias-Corrected Effectiveness of Traditional Chinese Medicine in Non-Small Cell Lung Cancer (C-EVID): A Prospective Cohort Study

**DOI:** 10.3389/fonc.2022.845613

**Published:** 2022-04-22

**Authors:** Xing Zhang, Qiujun Guo, Conghuang Li, Rui Liu, Tao Xu, Zhichao Jin, Yupeng Xi, Yinggang Qin, Weidong Li, Shuntai Chen, Ling Xu, Lizhu Lin, Kang Shao, Shenyu Wang, Ying Xie, Hong Sun, Ping Li, Xiangyang Chu, Kequn Chai, Qijin Shu, Yanqing Liu, Yue Zhang, Jiaqi Hu, Bolun Shi, Xiwen Zhang, Zhenhua Zhang, Juling Jiang, Shulin He, Jie He, Mingxi Sun, Ying Zhang, Meiying Zhang, Honggang Zheng, Wei Hou, Baojin Hua

**Affiliations:** ^1^ Department of Oncology, Guang’anmen Hospital, China Academy of Chinese Medical Sciences, Beijing, China; ^2^ Department of Oncology, Xiyuan Hospital of China Academy of Chinese Medicine, Beijing, China; ^3^ Department of Oncology, Jiangsu Province Hospital of Chinese Medicine, Nanjing, China; ^4^ Department of Oncology, Yueyang Hospital of Integrated Traditional Chinese and Western Medicine, Shanghai University of Traditional Chinese Medicine, Shanghai, China; ^5^ Department of Oncology, The First Affiliated Hospital of Guangzhou University of Chinese Medicine, Guangzhou, China; ^6^ Department of Thoracic Surgery, Cancer Hospital Chinese Academy of Medical Sciences, Beijing, China; ^7^ Department of Integrated Traditional Chinese Medicine (TCM) & Western Medicine, Liaoning Cancer Hospital & Institute, Shenyang, China; ^8^ Department of Traditional Chinese Medicine, Shanxi Provincial Cancer Hospital, Taiyuan, China; ^9^ Department of Integrated Traditional Chinese Medicine (TCM) & Western Medicine, Beijing Cancer Hospital, Beijing, China; ^10^ Department of Oncology, The First Affiliated Hospital of Anhui Medical University, Hefei, China; ^11^ Department of Thoracic Surgery, Chinese PLA General Hospital, Beijing, China; ^12^ Department of Traditional Chinese Medicine, Tongde Hospital of Zhejiang Province, Hangzhou, China; ^13^ Department of Oncology, Zhejiang Provincial Hospital of Chinese Medicine, Hangzhou, China; ^14^ Department of Combined Traditional Chinese and Western Medicine, Yangzhou University School of Medicine, Yangzhou, China; ^15^ Department of Integrated Traditional Chinese Medicine (TCM) & Western Medicine, Jilin Cancer Hospital, Changchun, China

**Keywords:** Chinese medicine, lung cancer, chemotherapy, prognosis, toxicity

## Abstract

**Background:**

Relatively little is known about the effect of traditional Chinese medicine (TCM) on prognosis of non-small cell lung cancer (NSCLC).

**Methods:**

In this nationwide, multicenter, prospective, cohort study, eligible patients aged 18-75 years with radical resection, and histologically confirmed stage II-IIIA NSCLC were enrolled. All patients received 4 cycles of standard adjuvant chemotherapy. Patients who received Chinese herbal decoction and (or) oral Chinese patent medicine for a cumulative period of not less than 6 months were defined as TCM group, otherwise they were considered as control group. The primary endpoint was DFS calculated using the Kaplan–Meier method. A time-dependent Cox proportional hazards model was used to correct immortal time bias. The secondary endpoints included DFS in patients of different characteristics, and safety analyses. This study was registered with the Chinese Clinical Trial Registry (ChiCTR1800015776).

**Results:**

A total of 507 patients were included (230 patients in the TCM group; 277 patients in the control group). The median follow-up was 32.1 months. 101 (44%) in the TCM group and 186 (67%) in the control group had disease relapse. The median DFS was not reached in the TCM group and was 19.4 months (95% CI, 14.2 to 24.6) in the control group. The adjusted time-dependent HR was 0.61 (95% CI, 0.47 to 0.78), equalling to a 39% reduction in the risk of disease recurrence with TCM. the number needed to treat to prevent one patient from relapsing was 4.29 (95% CI, 3.15 to 6.73) at 5 years. Similar results were observed in most of subgroups. Patients had a significant improvement in white blood cell decrease, nausea, decreased appetite, diarrhea, pain, and fatigue in the TCM group.

**Conclusion:**

TCM may improves DFS and has a better tolerability profile in patients with stage II-IIIA NSCLC receiving standard chemotherapy after complete resection compared with those receiving standard chemotherapy alone. Further studies are warranted.

## Introduction

Lung cancer is the leading cause of cancer death worldwide, with an estimated 1.8 million deaths in the year 2020 (1). Approximately 76% of cases are non-small cell lung cancer (NSCLC) (2). Surgical resection is the optimal treatment for stage II-IIIA NSCLC and adjuvant cisplatin-based chemotherapy is recommended for routine use in patients who have undergone radical surgical resections (3). A large number of studies performed since the 1990s have suggested the benefit of adjuvant chemotherapy for disease-free survival (DFS), however, with minimal absolute 5-year DFS benefits (4.3% to 5.8%) after complete resection in patients with early-stage NSCLC (4–6). The outcomes of these studies suggest that the development of adjuvant chemotherapy has plateaued during the past two decades, with no additional gains in DFS over this period. Moreover, the toxicity of chemotherapy has always been a concern for clinicians. The rate of overall grade 3 to 4 adverse events (AEs) of cisplatin-based chemotherapy varies from 66% to 90% across studies with different regimens and data collection (3, 6, 7). Otherwise, chemotherapy-related deaths (0.9%) have been reported in previous studies (3). Most patients receiving treatment with chemotherapy experienced hematologic and non-hematologic AEs, such as neutropenia, anemia, fatigue, nausea, anorexia, and vomiting (8), which decreased their quality of life and might lead to dose reduction, delays, or chemotherapy discontinuation. Minimal benefits and toxicity of chemotherapy in patients with early-stage NSCLC after complete resection emphasize that this treatment approach requires additional improvements.

Cancer patients in low- and middle-income countries are often treated with traditional, complementary, and integrative medicine that is more familiar, less costly, and widely available (9). Previous studies have reported that 21% up to nearly 90% of cancer patients in Asia used traditional Chinese medicine (TCM) (10, 11), while 25%-47% of Chinese cancer patients in the North America used TCM as part of their cancer treatments (12, 13). Studies have suggested that TCM could inhibit lung cancer cell growth, possibly by inhibiting nuclear factor kappa-B (NF-κB) activity (14, 15) (NF-κB promotes tumor progression, mainly by protecting transformed cells from apoptosis) (16). In addition, TCM can also enhance the immune function of patients by stimulating the activity of natural killer cells and facilitating the maturation of dendritic cells (17, 18). In clinical trials, TCM in combination with adjuvant chemotherapy, compared with adjuvant chemotherapy alone, has been shown to significantly improve symptoms such as vomiting, fatigue, pain, and dry mouth, as well as reduce hematologic AEs in early-stage NSCLC patients after complete resection (19, 20). Moreover, in these patients, TCM might reduce the risk of NSCLC recurrence.

Our previous study suggested that TCM in combination with conventional postoperative treatment was associated with better DFS [hazard ratio (HR), 0.42; 95% confidence interval (CI), 0.31 to 0.57], compared with conventional postoperative treatment alone, in stage I-IIIA NSCLC patients after complete resection (21). However, adjuvant chemotherapy is only recommended for routine use in patients with stage II-IIIA NSCLC (3). Therefore, we aimed to investigate whether TCM can reduce the risk of recurrence in patients with stage II-IIIA NSCLC receiving adjuvant chemotherapy after radical resection.

## Patients and Methods

### Study Design and Patients

C-EVID was a nationwide, multicenter, prospective, cohort study, conducted at 13 hospitals in 9 provinces and cities of China (Beijing, Shanghai, Guangdong, Liaoning, Jilin, Shanxi, Anhui, Zhejiang, and Jiangsu). Patients aged 18-75 years with histologically confirmed stage II-IIIA NSCLC [as classified according to the seventh edition of the Cancer Staging Manual of the American Joint Committee on Cancer (AJCC)], who underwent radical resection up to 3 months before included, were enrolled from November 10, 2014 to February 10, 2017. The final follow-up was conducted on April 15, 2021. Additional inclusion criteria were normal function of the heart, liver, kidney and hematopoietic system; Karnofsky physical status (KPS) ≥ 60; and informed consent. Exclusion criteria included pregnant women, patients with mental disorders, and a survival period of < 6 months.

This study was conducted in accordance with the principles of the Declaration of Helsinki. The study protocol was approved by the Medical Ethics Committee of Guang ‘anmen Hospital of Chinese Academy of Chinese Medical Sciences (2014EC108-05) and registered in the Chinese Clinical Trial Registry (ChiCTR1800015776).

### Study Treatment and Exposure

All enrolled patients received 4 cycles of first-line of adjuvant chemotherapy according to the National Comprehensive Cancer Network Clinical Practice Guideline for NSCLC (Version I, 2014). Our previous study suggested that NSCLC patients treated with TCM for 6 months had a better DFS (21). In this study, patients who received Chinese herbal decoction and (or) oral Chinese patent medicine for a cumulative period of not less than 6 months were defined as the TCM group (exposure group).

Patients, who did not receive Chinese herbal decoction and (or) oral Chinese patent medicine or received such treatments for less than 6 months, would be considered as control group (non-exposure group). The cumulative exposure time of TCM was calculated based on medical records.

### Assessments

The primary endpoint was the DFS. DFS was calculated from the time of enrolment until the time of disease recurrence or death from any cause. Disease recurrence was evaluated according to the Response Evaluation Criteria in Solid Tumors (RECIST) version 1.1 (determined by computed tomography, magnetic resonance imaging, and pathological biopsy) (22).

The secondary endpoints included DFS in patients of different sexes, ages, smoking histories, body mass indexes (BMIs), histologic types, AJCC stages, regional lymph nodes, degrees of differentiation, and adjuvant therapies. Safety analyses, which were classified based on the National Cancer Institute Common Terminology Criteria for Adverse Events (version 4.0), included hematologic and non-hematologic AEs during the chemotherapy period. The use of Chinese herbal decoction and oral Chinese patent medicine would be reported. Follow-ups were performed every 3 weeks during the chemotherapy period, then every 12 weeks for one year, every 24 weeks for two years, and yearly thereafter.

### Statistical Analysis

Baseline characteristics would be presented as the mean (standard deviation) or median (interquartile range) for continuous variables and as the number (percentage) for categorical variables. We would use t-test for continuous variables following normal distribution; otherwise, a nonparametric test would be performed. We would use the χ^2^ test or Fisher’s exact test for categorical variables.

DFS would be calculated using the Kaplan–Meier method and compared using the log-rank test. Immortal time bias is common in cohort studies of drug effects (23). A time-dependent Cox proportional hazards model with adjustments would be used to correct immortal time bias (23). The adjustments were for sex, age, smoking, BMI, histologic type, AJCC stage, regional lymph nodes, poor differentiation, KPS score, adjuvant therapy. Prespecified subgroup analyses would be performed in patients with different clinical characteristics and shown in a forest plot. A test of interaction was performed to explore the differential treatment effects between subgroups (24). AEs would be presented as a number (percentage) and calculated using the χ^2^ test. The numbers of cases treated by Chinese herbal decoction and (or) oral Chinese patent medicine would also be recorded. Statistical analyses were performed using SPSS Statistics 25 and R version 4.1.0. All tests were two-sided and a *P* value <0.05 was considered statistically significant.

## Results

From November 10, 2014 to February 10, 2017, a total of 513 patients were recruited. After excluding 6 patients (small cell lung cancer = 1, stage IV NSCLC = 3, without informed consent = 2, **Supplementary Figure 1**), this cohort study enrolled 507 patients (230 patients in the TCM group and 277 patients in the control group). The median follow-up was 32.1 months (interquartile range [IQR], 11.8 to 59.1). The mean duration of total treatment of the TCM group was 23.5 months (range, 6.0 to 73.5).

**Table 1** presents the baseline characteristics of patients according to the TCM and control groups. The median age was 58 years in the TCM group and was 59 years in the control group. There were 61.7% and 68.2% males in the TCM and control groups, respectively. The TCM and control groups were similar in terms of smoking history, BMI, AJCC stage, KPS score, poor differentiation. Compared with the control group, the TCM group had more patients with stage IIA NSCLC (30.4% vs 21.3%) and fewer patients receiving radiotherapy (14.8% vs 21.7%).

**Table 1:** Demographic and Clinical Characteristics of Patients at baseline.

Characteristic	TCM (n=230)	Control (n=277)	*P*
Age, median (IQR), y	58 (52, 63)	59 (52, 64)	0.11^a^
Sex			
Male	142 (61.7%)	189 (68.2%)	0.12
Female	88 (38.3%)	88 (31.8%)	
Smoking History			
Yes	121 (52.6%)	145 (52.3%)	0.95
No	109 (47.4%)	132 (47.7%)	
BMI, median (IQR), kg/m^2^	23.7 (22.0, 25.3)	23.2 (21.6, 25.2)	0.32^a^
Histologic type			
ADC	150 (65.2%)	174 (62.8%)	0.65
SCC	67 (29.1%)	85 (30.7%)	
ADSC	6 (2.6%)	12 (4.3%)	
Others	7 (3.0%)	6 (2.2%)	
AJCC stage			
IIa	70 (30.4%)	59 (21.3%)	0.04^b^
IIb	44 (19.1%)	61 (22.0%)	
IIIa	116 (50.4)	157 (56.7%)	
KPS score			
100	14 (6.1%)	13 (4.7%)	0.30^b^
90	178 (77.4%)	233 (84.1%)	
≤80	38 (16.5%)	31 (11.2%)	
Regional lymph nodes			
N0	45 (19.6%)	32 (11.6%)	0.18^b^
N1	84 (36.5%)	122 (44.0%)	
N2	101 (43.9%)	123 (44.4%)	
Poor differentiation			
Yes	73 (31.7%)	106 (38.3%)	0.13
No	157 (68.3%)	171 (61.7%)	
Radiotherapy			
Yes	34 (14.8%)	60 (21.7%)	<0.05
No	196 (85.2%)	217 (78.3%)	

IQR, interquartile range; TCM, traditional Chinese medicine; BMI, body mass index (calculated as weight in kilograms divided by height in meters squared); ADC, adenocarcinoma; SCC, squamous cell carcinoma; ADSC, adenosquamous carcinoma; AJCC, American Joint Committee on Cancer. ^a^Nonparametric test. ^b^χ2 test for trend.

**Figure 1:**
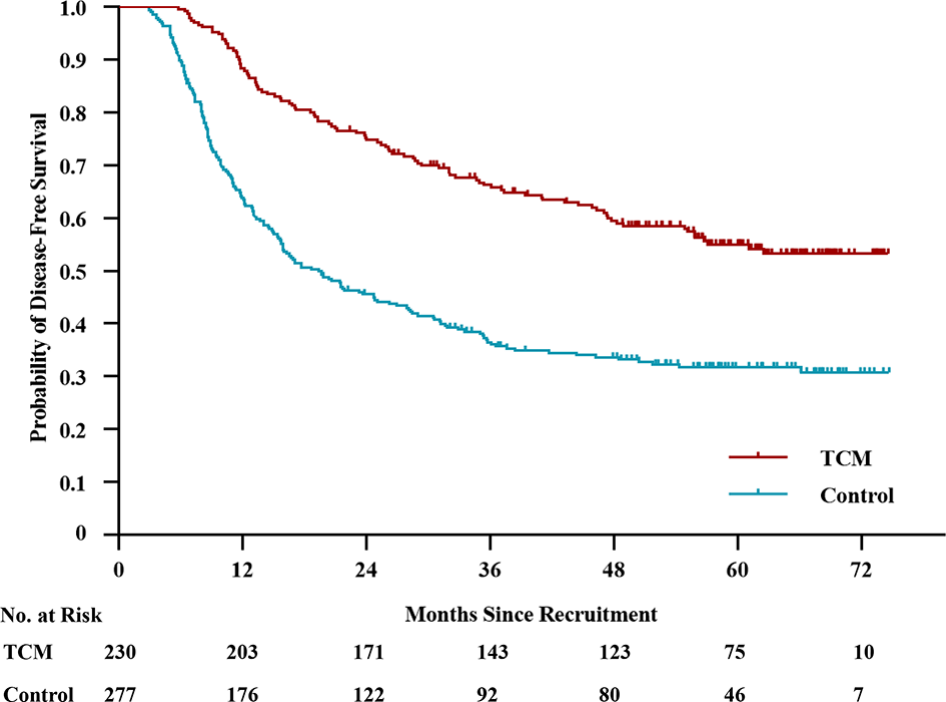
Kaplan–Meier Estimates of Disease-free Survival.

By the last follow-up, disease recurrence or death occurred in 287 patients. There were 101 and 186 events in the TCM and control groups, respectively. The median DFS was not reached in the TCM group and was 19.4 months (95% CI, 14.2 to 24.6) in the control group (**Figure 1**). The 2-year DFS rate was 74.8% (95% CI, 96.1% to 80.5%) in the TCM group and 45.6% (95% CI, 39.7% to 51.5%) in the control group. The 3-year DFS rate was 66.3% (95% CI, 60.2% to 72.4%) in the TCM group and 36.5% (95% CI, 30.8% to 42.2%) in the control group. The 5-year DFS rate was 55.1% (95% CI, 48.4% to 61.8%) in the TCM group and 31.8% (95% CI, 26.1% to 37.5%) in the control group (unadjusted HR, 0.46; 95% CI, 0.36 to 0.58; *P* < 0.01). Considering immortal time bias, a time-dependent Cox proportional hazards model was performed and the adjusted HR was 0.61 (95% CI, 0.47 to 0.78; *P* < 0.01). This HR, which was equal to a 39% reduction in the risk of disease recurrence or death, and the number needed to treat to prevent one patient from relapsing was 4.29 (95% CI, 3.15 to 6.73) at 5 years, indicating that patients in the TCM group had a significantly longer DFS than those in the control group.

**Figure 2:**
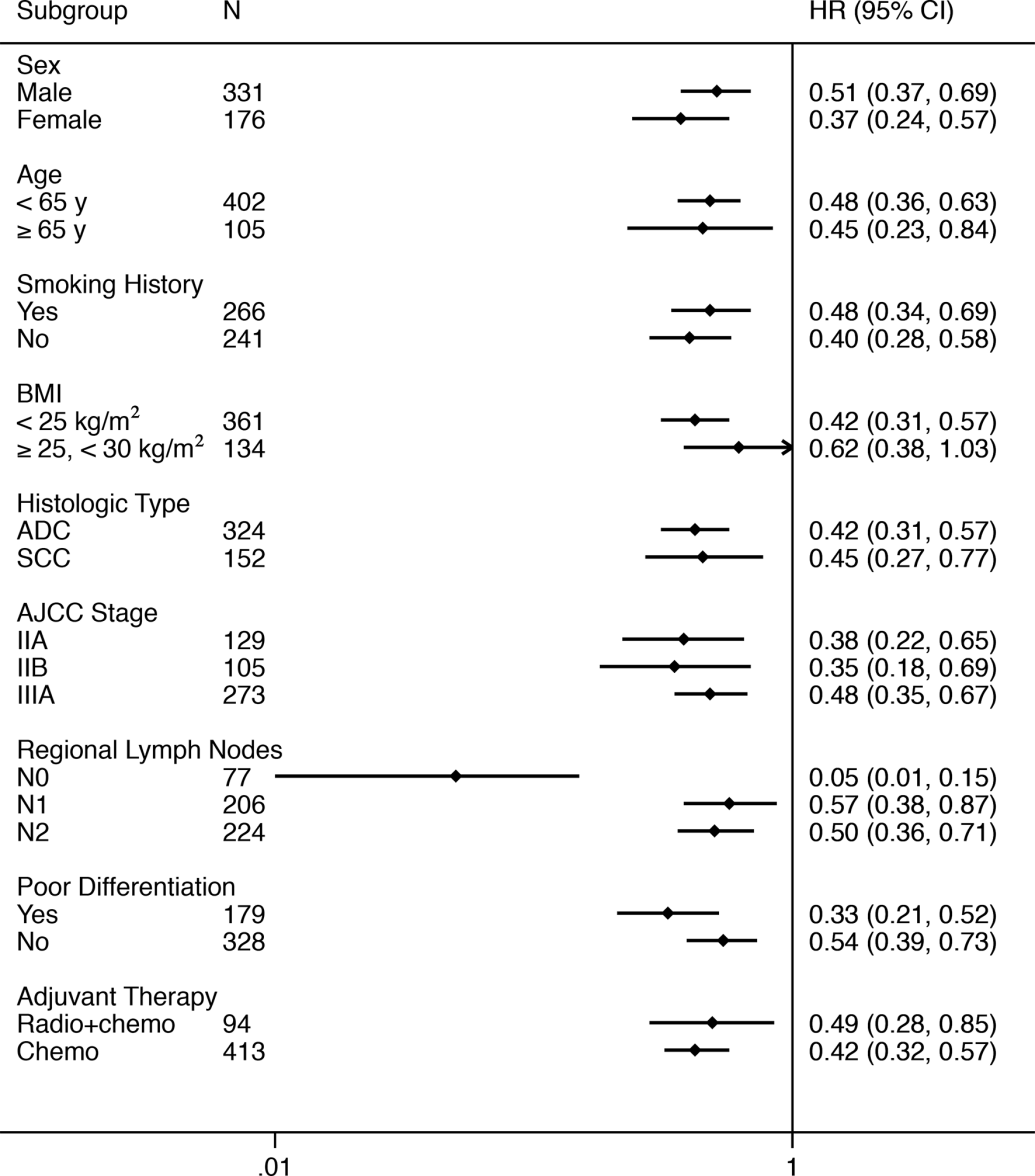
Forest Plot of the Treatment Effect on Disease-free Survival in Subgroup Analyses. BMI, body mass index (calculated as weight in kilograms divided by height in meters squared); ADC, adenocarcinoma; SCC, squamous cell carcinoma; AJCC, American Joint Committee on Cancer; Radio, radiotherapy; Chemo, chemotherapy.

The benefit favoring TCM with respect to DFS was observed consistently in most of predefined subgroups (**Table 2** and **Figure 2**). There was a 49% (HR, 0.51; 95% CI, 0.37 to 0.69; *P* < 0.01) and 63% (HR, 0.37; 95% CI, 0.24 to 0.57; *P* < 0.01) reduction in the risk of disease recurrence or death in patients who received TCM for males and females, respectively. In the subgroup analysis of histologic type, all patients with adenocarcinoma (HR, 0.42; 95% CI, 0.31 to 0.57; *P* < 0.01) and squamous cell carcinoma (HR, 0.45; 95% CI, 0.27 to 0.77; *P* < 0.01) in the TCM group had a significantly longer DFS than those in the control group. TCM also showed a significantly longer DFS compared with standard chemotherapy alone in patients with stage IIA NSCLC (HR, 0.38; 95% CI, 0.22 to 0.65; *P* < 0.01, **Supplementary Figure 2**), stage IIB NSCLC (HR, 0.35; 95% CI, 0.18 to 0.69; *P* < 0.01, **Supplementary Figure 3**), and stage IIIA NSCLC (HR, 0.48; 95% CI, 0.35 to 0.67; *P* < 0.01, **Supplementary Figure 4**), respectively. Similar results were observed in the subgroup analyses of adjuvant therapy. The HRs was 0.49 (95% CI, 0.28 to 0.85; *P* = 0.01) for radiotherapy + chemotherapy, and 0.42 (95% CI, 0.32 to 0.59; *P* < 0.01) for chemotherapy. As for regional lymph nodes, the HRs was 0.05 (95% CI, 0.01 to 0.15; *P* < 0.01) for stage N0, 0.57 (95% CI, 0.38 to 0.87; *P* < 0.01) for stage N1, and 0.50 (95% CI, 0.36 to 0.71; *P* < 0.01) for stage N2. A test of interaction between regional lymph nodes and TCM treatments on DFS was statistically significant (*P* < 0.05).

**Table 2:** Subgroup Analysis of Disease-free Survival.

Subgroup	No. of patients	HR (95%CI)	*P*	*P_interaction_*
Sex				0.21
Male	331	0.51 (0.37, 0.69)	<0.01	
Female	176	0.37 (0.24, 0.57)	<0.01	
Age				0.46
<65 y	402	0.48 (0.36, 0.63)	<0.01	
≥65 y	105	0.45 (0.23, 0.84)	0.01	
Smoking History				0.47
Yes	266	0.48 (0.34, 0.69)	<0.01	
No	241	0.40 (0.28, 0.58)	<0.01	
BMI				0.18
<25 kg/m^2^	361	0.42 (0.31, 0.57)	<0.01	
≥25, <30 kg/m^2^	134	0.62 (0.38, 1.03)	0.06	
Histologic type				0.97
ADC	324	0.42 (0.31, 0.57)	<0.01	
SCC	152	0.45 (0.27, 0.77)	<0.01	
AJCC stage				0.59
IIA	129	0.38 (0.22, 0.65)	<0.01	
IIB	105	0.35 (0.18, 0.69)	<0.01	
IIIA	273	0.48 (0.35, 0.67)	<0.01	
Regional lymph nodes				<0.05
N0	77	0.05 (0.01, 0.15)	<0.01	
N1	206	0.57 (0.38, 0.87)	<0.01	
N2	224	0.50 (0.36, 0.71)	<0.01	
Poor differentiation				0.17
Yes	179	0.33 (0.21, 0.52)	<0.01	
No	328	0.54 (0.39, 0.73)	<0.01	
Adjuvant therapy				0.39
Radiotherapy+chemotherapy	94	0.49 (0.28, 0.85)	0.01	
Chemotherapy	413	0.42 (0.32, 0.57)	<0.01	

BMI, body mass index (calculated as weight in kilograms divided by height in meters squared); ADC, adenocarcinoma; SCC, squamous cell carcinoma; AJCC, American Joint Committee on Cancer. Adjusted by sex, age, smoking history, BMI, histologic type, AJCC stage, regional lymph nodes, poor differentiation, and adjuvant therapy. P value for interaction test: 2-way interaction of clinical characteristics and treatment groups (traditional Chinese medicine and control group) on disease-free survival.

AEs during the chemotherapy period are shown in **Table 3**. During the chemotherapy period, only patients with TCM exposure in the TCM group and those without TCM exposure in the control group were included in the analyses of AEs. White blood cell count decreased in fewer patients (53%) in the TCM group than in the control group (68%) (*P* = 0.02). Similarly, fewer patients experienced fatigue, pain, decreased appetite, nausea, diarrhea in the TCM group than those in the control group (*P* < 0.05).

**Table 3:** Adverse Events.

Adverse event	TCM (N=192)					Control (N=227)					*P^a^*
	Any Grade	Grade 1	Grade 2	Grade 3	Grade 4	Any Grade	Grade 1	Grade 2	Grade 3	Grade 4
	Number of patients (percent)										
White blood cell decreased	102 (53)	52 (27)	45 (23)	1 (<1)	4 (2)	154 (68)	63 (28)	64 (28)	19 (8)	8 (4)	0.02
Neutrophil count decreased	113 (59)	63 (33)	29 (15)	16 (8)	5 (3)	169 (74)	92 (41)	50 (22)	16 (7)	11 (5)	0.94
Anemia	99 (52)	71 (37)	21 (11)	3 (2)	3 (2)	147 (65)	98 (43)	39 (17)	3 (1)	7 (3)	0.39
Platelet count decreased	34 (18)	22 (12)	5 (3)	3 (2)	4 (2)	65 (29)	47 (21)	8 (4)	3 (1)	7 (3)	0.52
Alanine aminotransferase increased	40 (21)	28 (15)	6 (3)	0	6 (3)	67 (30)	43 (19)	10 (4)	3 (1)	11 (5)	0.56
Aspartate aminotransferase increased	26 (14)	18 (9)	2 (1)	0	6 (3)	48 (21)	32 (14)	5 (2)	0	11 (5)	0.94
Gamma-glutamyltransferase increased	29 (15)	19 (10)	3 (2)	0	7 (4)	76 (33)	48 (21)	11 (5)	3 (1)	14 (6)	0.85
Fatigue	170 (89)	130 (68)	34 (18)	6 (3)	/	221 (97)	85 (37)	129 (57)	7 (3)	/	<0.01
Weight loss	61 (32)	56 (29)	4 (2)	1 (<1)	/	107 (47)	101 (45)	6 (3)	0	/	0.35
Pain	60 (31)	58 (30)	2 (1)	0	/	122 (54)	106 (47)	16 (7)	0	/	0.04
Decreased appetite	160 (83)	129 (67)	29 (15)	2 (1)	0	215 (95)	125 (55)	86 (38)	4 (2)	0	<0.01
Constipation	51 (27)	45 (23)	4 (2)	2 (1)	0	66 (29)	57 (25)	9 (4)	0	0	0.78
Dry mouth	101 (53)	99 (52)	2 (1)	0	/	145 (64)	135 (60)	10 (4)	0	/	0.08
Nausea	151 (79)	123 (64)	28 (15)	0	/	207 (91)	136 (60)	66 (29)	5 (2)	/	<0.01
Vomiting	65 (34)	54 (28)	10 (5)	1 (<1)	0	130 (57)	106 (47)	22 (10)	2 (<1)	0	0.82
Diarrhea	29 (15)	24 (13)	4 (2)	1 (<1)	0	44 (19)	44 (19)	0	0	0	<0.01
Alopecia	91 (47)	67 (35)	24 (13)	/	/	126 (56)	82 (36)	44 (19)	/	/	0.18
Pruritus	32 (17)	30 (16)	2 (1)	0	/	101 (44)	93 (41)	7 (3)	1 (<1)	/	0.67
Rash acneiform	39 (20)	39 (20)	0	0	0	81 (36)	78 (34)	2 (1)	1 (<1)	0	0.25

TCM, traditional Chinese medicine. ^a^χ^2^ test for trend.

**Figure 3:**
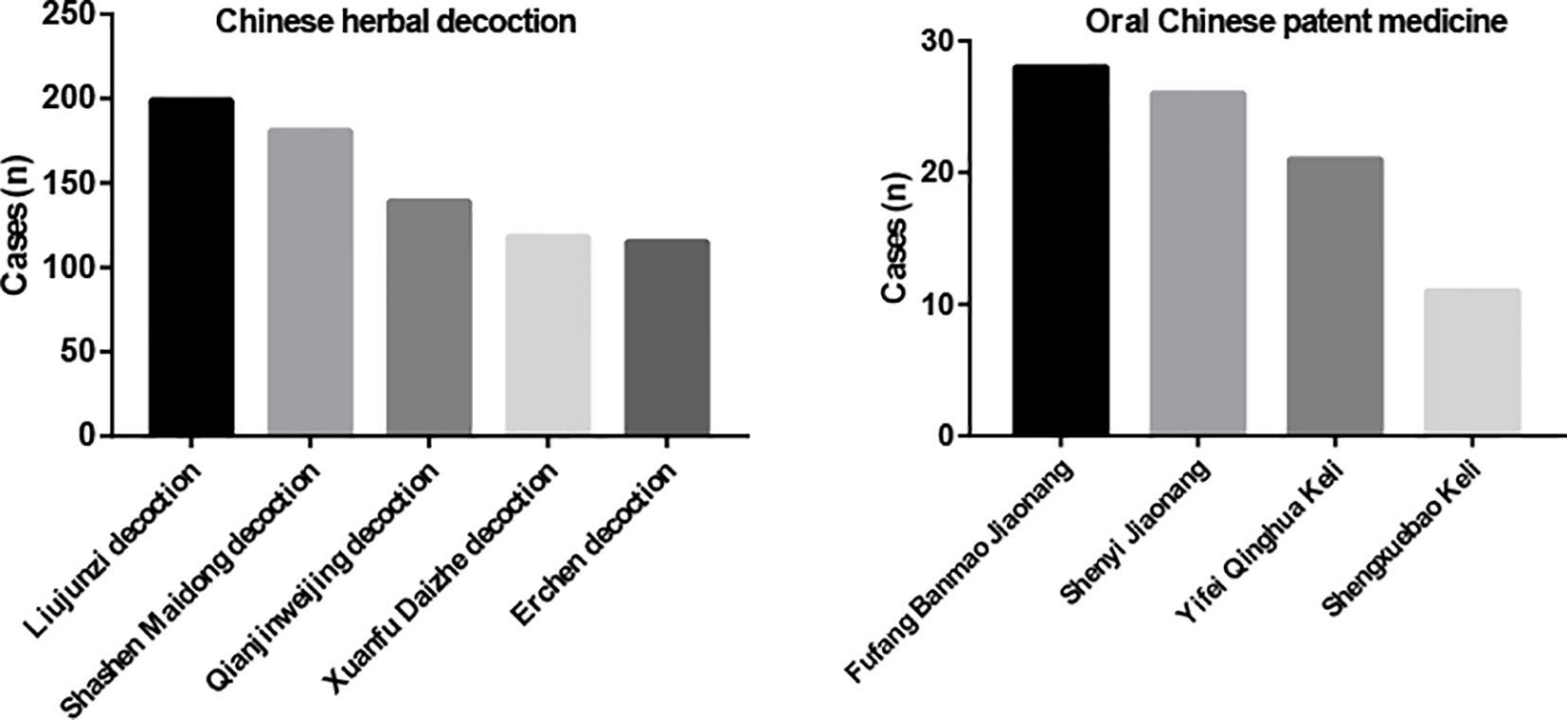
Use of Chinese Herbal Medicine and Oral Chinese Patent Medicine.

The five most commonly used Chinese herbal decoctions were Liujunzi decoction (199 cases, 87%), Shashen Maidong decoction (181 cases, 79%), Qianjinweijing decoction (139 cases, 60%), Xuanfu Daizhe decoction (118 cases, 51%), and Erchen decoction (115 cases, 50%) (**Figure 3**). As for oral Chinese patent medicine, Fufang Banmao Jiaonang (28 cases, 12%), Shenyi Jiaonang (26 cases, 11%), Yifei Qinghua Keli (21 cases, 9%), and Shengxuebao Keli (11 cases, 5%) were used more commonly. (**Figure 3**) The ingredients of Chinese herbal decoctions and oral Chinese patent medicines are shown in **Supplementary Table 1**.

In the TCM group, all patients were treated with “Fuzheng” medicine, which includes drugs that have the function of strengthening “Zang and Fu”, tonifying Qi, replenishing blood, nourishing Yin, and tonifying Yang (25). Cytotoxic TCM with antitumor effect, according to pharmacological research, were also widely used in our study. A *post hoc* analysis was performed to explore the effectiveness in different TCM treatments in the TCM group. Patients who received TCM treatments containing cytotoxic components with antitumor effect, would be considered as “cytotoxic + Fuzheng” group, and the rest were considered as “Fuzheng” group. The adjusted HR was 0.81 (95% CI, 0.53 to 1.25; P = 0.34) (**Table 4**).

**Table 4:** Subgroup Analysis of Disease-free Survival in TCM Group.

Subgroup	No. of patients	HR (95% CI)	*P*
Fuzheng	142	1	0.34
Fuzheng+cytotoxic	88	0.81 (0.53, 1.25)	

Adjusted by sex, age, smoking history, BMI, histologic type, AJCC stage, regional lymph nodes, poor differentiation, and radiotherapy.

## Discussion

In this nationwide, multicenter, prospective, cohort study, TCM significantly improved DFS in patients with stage II-IIIA NSCLC receiving standard chemotherapy after complete resection compared with patients who received standard chemotherapy alone. With respect to the primary endpoint, 44% of patients in the TCM group and 67% of patients in the control group relapsed or died (adjusted HR, 0.61; 95% CI, 0.47 to 0.78; *P* < 0.01), equalling to a 39% reduction in the risk of disease recurrence or death with TCM. With respect to the secondary endpoints, the DFS benefit was observed consistently in most of predefined subgroups, including subgroups defined by sex, age, smoking history, histologic type, AJCC stage, regional lymph nodes, poor differentiation, and adjuvant therapy. In our study, adjuvant therapy included chemotherapy (for all patients) and radiotherapy (only for parts of IIIA patients). To explore which one getting best from TCM, we did the interaction test. Unfortunately, we could not conclude which one got best from TCM (*P_interaction_* = 0.39). Interestingly, the benefit of TCM in patients with stage N0 was significantly better than that in patients with stage N1 and N2 (*P_interaction_* < 0.05). Considering that this was an exploratory analysis and the sample size was small, we should be cautious in interpreting this result. Fewer patients in the TCM group experienced hematologic and non-hematologic adverse events including white blood cell decrease, fatigue, pain, decreased appetite, nausea, diarrhea than patients in the control group during the chemotherapy period (*P* < 0.05).

Adjuvant chemotherapy is recommended for routine use in patients with completely resected, stage II-IIIA NSCLC. In our study, more patients in the control group relapsed than those in the TCM group, and the DFS rate is consistent with historical data in stage II-IIIA NSCLC patients receiving adjuvant chemotherapy (26–29). The efficacy of adjuvant chemotherapy has stabilized during the past two decades, highlighting the need for more effective adjuvant treatment options (30). TCM, a potentially effective adjuvant therapy for NSCLC, has been commonly used in China. Recently, a study has shown a similar DFS benefit with TCM (unadjusted HR, 0.41; 95% CI, 0.20 to 0.84) in patients with completely resected stage II-IIIA NSCLC after standard chemotherapy compared with patients who received standard chemotherapy only (31). However, this study was a preliminary retrospective study with a small sample size, and a more rigorous study was needed to confirm this result.

Our previous study, which included postoperative patients with stage I-IIIA, found a significantly longer DFS (HR, 0.42; 95% CI, 0.31 to 0.57) in patients receiving continuous TCM treatment compared with those receiving conventional postoperative treatment (21). In the exploratory analysis, we found that TCM might also be a protective factor for recurrence in stage II-IIIA NSCLC patients (21). Considering that adjuvant chemotherapy is not recommended in patients with stage IA NSCLC and not recommended for routine use in patients with stage IB NSCLC (3), our study did not include patients with stage I NSCLC. Clinically, some patients with NSCLC do not take Chinese medicine continuously but take it intermittently. To better reflect the real situation, the exposure time was calculated by the cumulative time of TCM rather than by the continuous time in our study.

The profile and severity of AEs of adjuvant chemotherapy recorded in our study are consistent with those recorded in previous studies (26, 28, 29). The most common hematologic AE was neutrophil count decrease (74%), followed by white blood cell decrease (68%) and anemia (65%) in patients receiving adjuvant chemotherapy alone. In the TCM group, significantly fewer patients experienced white blood cell decrease (53%, *P* = 0.02), and fewer patients, without statistical significance, experienced neutrophil count decrease (59%) and anemia (52%). Grade 3 or worse AEs in the TCM group were also less than those in the control group. In previous studies, gastrointestinal side effects were the most common non-hematologic AEs in patients receiving chemotherapy (26, 28). In our study, besides gastrointestinal side effects, we found that 97% of patients in the control group felt fatigued. In a randomized phase III study, fatigue was observed in approximately 60% of patients with completely resected stage II-IIIA nonsquamous NSCLC receiving vinorelbine plus cisplatin and in approximately 50% of patients receiving pemetrexed plus cisplatin (28). This difference might be due to patient heterogeneity and different study designs. Similarly, our study also suggested the benefit of TCM for non-hematologic toxicity of chemotherapy, especially on nausea, decreased appetite, diarrhea, pain, and fatigue (*P* < 0.05). These results were consistent with a previous randomized, double-blinded trial (19).

To our knowledge, our study is the first nationwide, multicenter, prospective, cohort study to evaluate the benefit of TCM on DFS in patients with completely resected, stage II-IIIA NSCLC. Although favorable results were observed in all patients and in most of predefined subgroups, several aspects should be considered when interpreting our outcomes. First, unlike randomized controlled trials, the baseline characteristics of the exposure and non-exposure groups in cohort studies may be different. In our study, the TCM group had more patients (30.4% vs 21.3%) with stage IIA NSCLC, suggesting a better prognosis. More patients (21.7% vs 14.8%) in the control group received radiotherapy, suggesting that these patients might have more risk factors (mediastinal lymph node metastasis, etc). The two factors might result in overestimation of the effectiveness of TCM. Two measures were employed to reduce the impact of baseline imbalance. One of the measures was that the primary outcome was adjusted by all baseline characteristics including stage and radiotherapy. The other one was subgroup analysis. In the subgroup analysis of AJCC stage, we found that all stages of NSCLC patients receiving TCM had significantly longer DFS than those receiving adjuvant chemotherapy (IIA: HR, 0,38; 95% CI, 0.22 to 0.65; IIB: HR, 0.35; 95% CI, 0.18 to 0.69; IIIA: 0.48; 95% CI, 0.35 to 0.67). Similar results were observed in subgroup analysis of radiotherapy. These analyses indicated the robustness of our results.

Second, immortal time bias is common in cohort studies of drug effects (23). Immortal time in cohort studies can bias the results in favor of the treatment group because only patients who survive for a long time can receive a treatment for a long time (23, 32). To obtain more reliable results of TCM on DFS, a time-dependent Cox proportional hazards model with adjustments was performed to remove immortal time bias (23, 33). A favorable benefit of TCM on DFS in postoperative patients with stage II-IIIA NSCLC was observed (HR, 0.61; 95% CI, 0.47 to 0.78; *P* < 0.01). Moreover, in the *post hoc* analysis, the power of our results was 99.9%. Additionally, self-selection bias could not be avoided in observational studies. Health-conscious patients in our study might be more likely to receive TCM treatment, which might lead to an overestimation of the protective effect of TCM.

Third, the goal of our study was to assess the efficacy of overall TCM (including Chinese herbal decoction and oral Chinese patent medicine) on DFS in postoperative patients with stage II-IIIA NSCLC receiving chemotherapy. “Fuzheng” and cytotoxic drugs were widely used in our study. A *post hoc* analysis was performed to explore the effectiveness in different TCM treatments in the TCM group. Based on current data, we could not conclude whether the “cytotoxic + Fuzheng” group benefits more or the “Fuzheng” group benefits more (adjusted HR, 0.81; 95% CI, 0.53 to 1.25; P = 0.34). In clinical practice, the TCM treatments vary according to patients’ stages of disease, symptoms, and performance status. It is difficult to draw a conclusion on which TCM ingredients play a major role in our study. Astragalus may have contributed greatly to our results. A pooled study reported that astragalus-based Chinese herbal medicine might increase the effectiveness (increase survival, tumor response, and performance status) of platinum-based chemotherapy compared with chemotherapy alone in advanced NSCLC patients (34). A previous study found that Astragalus polysaccharides, the active ingredient in Astragalus, could enhance the M1 polarization of macrophages, functional maturation of dendritic cells, and T cell-mediated anticancer immune responses in patients with lung cancer (35). Astragalus polysaccharides might ameliorate cancer-related fatigue and improve the quality of life of patients with metastatic disease according to the modulation of the inflammatory cascade (36, 37). 20 (R)-Ginsenoside Rg3, an active monomer extracted from ginseng, might sensitize hypoxic lung cancer cells to cisplatin by blocking of NF-κB mediated epithelial-mesenchymal transition and stemness (38). Considering the important role of TCM in strengthening the efficacy of adjuvant chemotherapy, we are conducting further studies to explore the possible effective ingredients of TCM in NSCLC.

Our study has several limitations. This study was conducted in China. Therefore, it is not clear whether our results are generalizable to other populations. Additionally, the median DFS was not reached in the TCM group. This might result from self-selection bias. The TCM group might include more health-conscious patients, which led to a long median DFS. Besides, insufficient follow-up time might be another reason for this result. However, the follow-up time in our study was the longest among similar studies (21, 31). The follow-up will continue, and the median DFS and survival curves will be updated in future analyses. A further limitation of our study is that some patients in the control group began to receive TCM treatment after disease recurrence, and these patients who met the exposure time would be divided into the TCM group. Thus, the grouping of overall survival data was different from that of DFS data. Considering that our primary endpoint was DFS, this study only reported the results of DFS, and the DFS is a valid surrogate for overall survival in studies of adjuvant setting (26). Finally, although a wide variety of covariates has been adjusted, as an observational study, residual confounding by unidentified confounders is still possible.

## Conclusion

In conclusion, this nationwide, multicenter, prospective, cohort study indicated that TCM may improves DFS and has a better tolerability profile in patients with stage II-IIIA NSCLC receiving standard chemotherapy after complete resection compared with patients receiving standard chemotherapy alone. Further studies are warranted to confirm our results and to elucidate the underlying mechanisms.

## Data Availability Statement

The original contributions presented in the study are included in the article/**Supplementary Material**. Further inquiries can be directed to the corresponding authors.

## Ethics Statement

The study involving human participants were reviewed and approved by the Medical Ethics Committee of Guang ‘anmen Hospital of Chinese Academy of Chinese Medical Sciences (2014EC108-05). The patients provided their written informed consent to participate in the study.

## Author Contributions

Conception and design: BH, WH, HZ. Provision of study material or patients: All authors. Collection and assembly of data: All authors. Data analysis and interpretation: XinZ, QG, CL, RL, TX, ZJ, YuX, BH, WH, HZ. Manuscript writing: XinZ. All authors read and approved the final manuscript.

## Funding

This study was supported by the National Twelfth Five-Year Plan for Science and Technology Support Program of China (2014BAI10B01), the National Natural Science Foundation of China (81774294, 82174465, 82174463, 82104961, 81904196), the Fundamental Research Funds for the Central public welfare research institutes (ZZ13-YQ-028, ZZ14-YQ-016), CACMS Innovation Fund (CI2021A01814, CI2021A01816).This study was also supported by Scientific and Technological Innovation Project of China Academy of Chinese Medical Sciences (CI2021B009) and Chinese Medicine Innovation Team and Talent Support Project (ZYYCXTD-C-202205).

## Conflict of Interest

The authors declare that the research was conducted in the absence of any commercial or financial relationships that could be construed as a potential conflict of interest.

## Publisher’s Note

All claims expressed in this article are solely those of the authors and do not necessarily represent those of their affiliated organizations, or those of the publisher, the editors and the reviewers. Any product that may be evaluated in this article, or claim that may be made by its manufacturer, is not guaranteed or endorsed by the publisher.
