# Supplementary material for: Immortal Time Bias-Corrected Effectiveness of Traditional Chinese Medicine in Non-Small Cell Lung Cancer (C-EVID): A Prospective Cohort Study
Source: Front Oncol. 2022 Apr 22;12:845613. doi: 10.3389/fonc.2022.845613 (PMC9076129; doi:10.3389/fonc.2022.845613)
Supplement: Supplementary file 1 [file DataSheet_1.docx]

Supplementary Material

**1 Supplementary Table 1** Ingredients of Traditional Chinese Medicine

| **Category** | **Drugs** | **Ingredients** |
| --- | --- | --- |
| Chinese herbal decoction | Liujunzi decoction | *Radix et rhizome ginseng, Rhizoma atractylodis macrocephalae, Poria, Radix et rhizoma glycyrrhizae praeparata cum melle, Pericarpium citri reticulatae, Rhizoma pinelliae* |
|  | Shashen Maidong decoction | *Radix glehniae, Rhizoma polygonati odorati, Radix ophiopogonis, Radix trichosanthis, Semen lablab album, Folium mori, Radix et rhizoma glycyrrhizae* |
|  | Qianjinweijing decoction | *Rhizoma phragmitis, Semen coicis, Semen persicae, Semen benincasae* |
|  | Xuanfu Daizhe decoction | *Flosinulae, Hematite, Rhizoma pinelliae, Radix et rhizoma ginseng, Radix et rhizoma glycyrrhizae praeparata cum melle, Rhizoma zingiberis recens, Fructus jujubae* |
|  | Erchen decoction | *Rhizoma pinelliae, Pericarpium citri reticulatae, Poria, Radix et rhizoma glycyrrhizae praeparata cum melle, Rhizoma zingiberis recens, Fructus mume* |
| Chinese patent medicine | Fufang Banmao Jiaonang | *Mylabris, Radix et rhizoma ginseng, Radix astragali, Radix et rhizoma seu caulis acanthopanacis senticosi, Rhizoma sparganii, Herba scutellariae barbatae, Rhizoma curcumae, Fructus corni, Fructus ligustri lucidi, Pulvis fellis ursi, Radix et rhizoma glycyrrhizae* |
|  | Shenyi Jiaonang | *Ginsenoside Rg3* |
|  | Yifei Qinghua Keli | *Radix astragali, Radix codonopsis, Radix glehniae, Radix ophiopogonis, Herba agrimoniae, Rhizoma bistortae, Herba patriniae, Hedyotis diffusa, Bulbus fritillariae cirrhosae, Radix et rhizoma asteris, Radix platycodonis, Semen armeniacae amarum, Radix et rhizoma glycyrrhizae* |
|  | Shengxuebao Keli | *Radix polygoni multiflori praeparata cum succo glycines sotae, Fructus ligustri lucidi, Fructus mori, Herba ecliptae, Radix paeoniae alba, Radix astragali, Rhizoma cibotii* |

## 2 Supplementary Figures
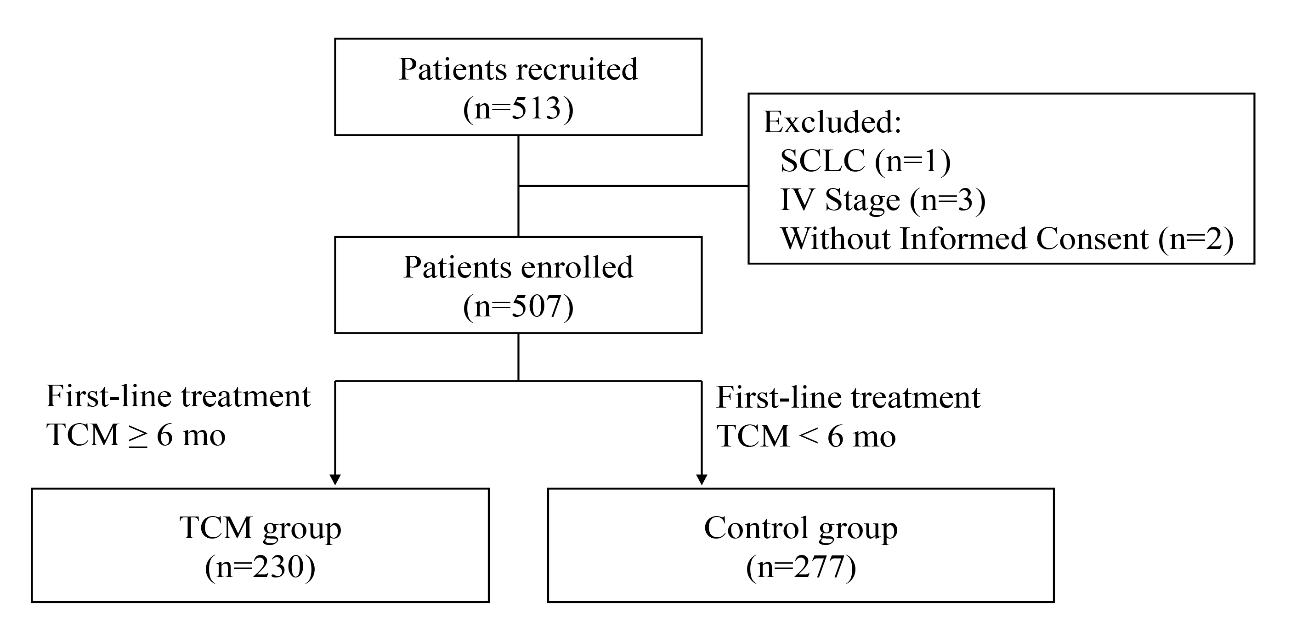
Supplementary Figure 1. Flow diagram.


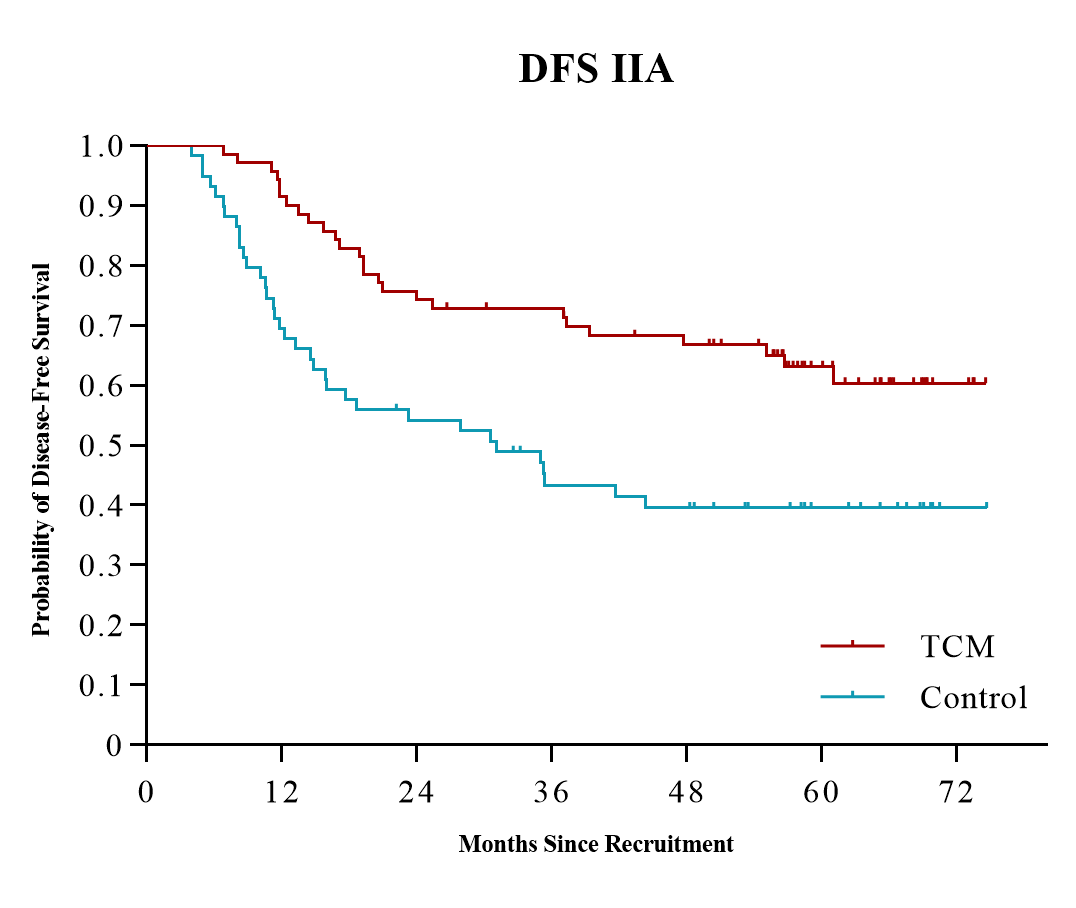


**Supplementary Figure 2** Kaplan–Meier Estimates of Disease-free Survival in Non-Small Cell Lung Cancer with Stage IIA


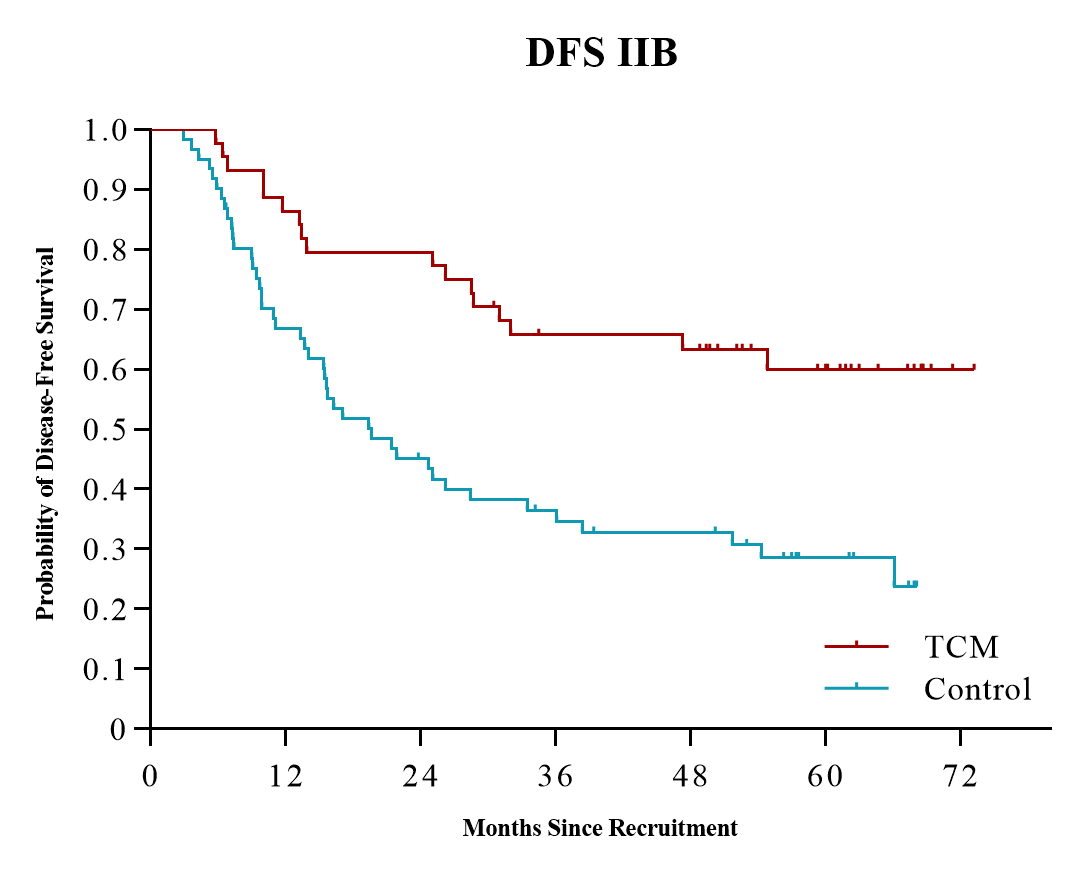


**Supplementary Figure 3** Kaplan–Meier Estimates of Disease-free Survival in Non-Small Cell Lung Cancer with Stage IIB


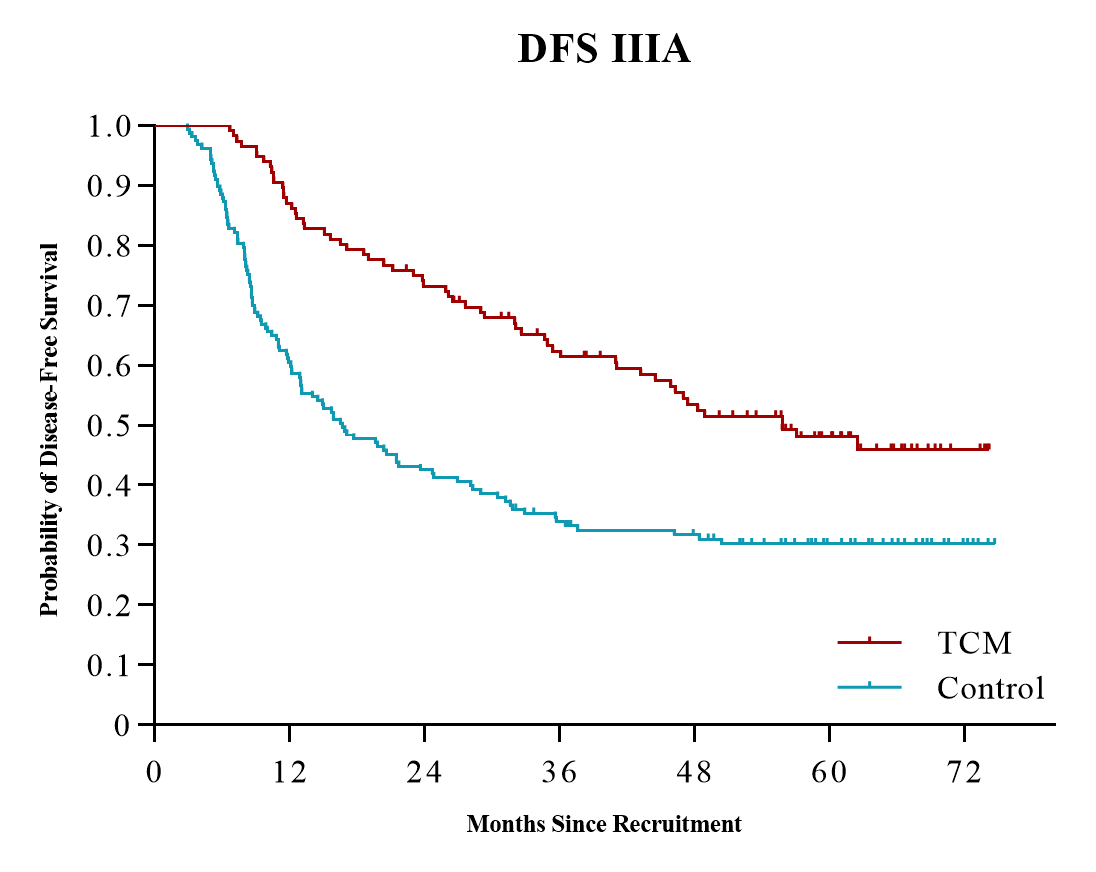


**Supplementary Figure 4** Kaplan–Meier Estimates of Disease-free Survival in Non-Small Cell Lung Cancer with Stage IIIA
